# Fast Polynomial Time Approximate Solution for 0-1 Knapsack Problem

**DOI:** 10.1155/2022/1266529

**Published:** 2022-10-22

**Authors:** Zhengyuan Wang, Hui Zhang, Yali Li

**Affiliations:** Xi'an Research Institute of Hi-Tech, Xi'an, Shaanxi 710025, China

## Abstract

0-1 Knapsack problem (KP) is NP-hard. Approximate solution is vital for solving KP exactly. In this paper, a fast polynomial time approximate solution (FPTAS) is proposed for KP. FPTAS is a local search algorithm. The best approximate solution to KP can be found in the neighborhood of the solution of upper bound for exact *k*-item knapsack problem (E-*k*KP) where *k* is near to the critical item *s*. FPTAS, in practice, often achieves high accuracy with high speed in solving KP. The computational experiments show that the approximate algorithm for KP is valid.

## 1. Introduction

0-1 Knapsack problem (KP) is a classical combinatorial optimization problem. KP is NP-hard. Knapsack problems were used to model capital budgeting problems in which investment projects are to be selected subject to expenditure limitations. Additionally, the knapsack problem has been used to model loading problems. Knapsack problems, moreover, arise as suboptimization problems in solving larger optimization problem [1]. KP can be formulated as follows:

(1) $$\begin{array}{c} \max\;f\left(x\right)=\overset{n}{\underset{i=1}{\sum }}p_{i}x_{i}\text{,}\\ s.t,\\ \overset{n}{\underset{i=1}{\sum }}w_{i}x_{i}\leq c\text{,}\\ x_{i}=0,1.i=1,2,\cdots ,n\text{,} \end{array}$$

where *p*_*i*_ is the profit of item *i*, *w*_*i*_ is the weight of item *i*, *c* is the capacity of the knapsack, *n* is the number of items in KP, and variable *x*_*i*_ = 0 or 1 indicates whether item *i* is selected or not. Without loss of generality, it is assumed that all items are arranged in nonincreasing order of efficiency.

(2) $$\begin{array}{c} \left(\frac{p_{i}}{w_{i}}\right)\geq \left(\frac{p_{i+1}}{w_{i+1}}\right)\left(i=1,2,\cdots ,n-1\right)\text{.} \end{array}$$

It is very important to find a fast polynomial approximation for KP in practice. Greedy algorithm [1], an approximate algorithm for KP, is on the basis of certain rules, such as the item being selected with priority over larger efficiency, larger profit, or smaller weight. Greedy algorithm is *O*(*n*) time complexity, but it is not an *ɛ*-approximate algorithm [1]. Approximation algorithm is often *k*-neighborhood local search method. Increasing the radius *k* of the neighborhood can improve the accuracy of the approximate algorithm [1, 2]. While PTAS for KP typically require only *O*(*n*) storage, all FPTAS are based on dynamic programming and their memory requirement increases rapidly with the accuracy *ɛ*, which makes them impractical even for relatively big values of *ɛ* [3]. Heuristics rules are adopted to decrease the calculation in searching accurate approximation, such as harmony search algorithm [4, 5], amoeboid organism algorithm [6], cuckoo search algorithm [5, 7], binary monarch butterfly optimization [8], cognitive discrete gravitational search algorithm [9], bat algorithm [10], and wind driven optimization [11]. Nowadays, it is tend to combine different heuristics together in solving combinatorial optimization problem, such as mixed-variable differentiate evolution [12], self-adaptive differential evolution algorithm [13], two-stage cooperative evolutionary algorithm [14], cooperative water wave optimization algorithm with reinforcement learning [15], and cooperative multi-stage hyper-heuristic algorithm [16]. However, these algorithms cannot guarantee the accuracy of the solution of KP. Meanwhile, these methods for the solution to KP are generally time-consuming. More domain knowledge is necessary for better algorithm. Pisinger gave an exact algorithm for KP [17] which is based on an expanding core. The items not included in the core are certain to be selected or not in the optimal solution, while the items in the core are uncertain to be selected or not in the optimal solution. He found that algorithms solving some kinds of core problem may be stuck by difficult cores [18]. For example, KP is determined by

(3) $$\begin{array}{c} w_{i}=i,p_{i}=10i^{2}+10^{7},c=200020,i=1,2,\cdots ,10^{4}\text{.} \end{array}$$

It is easy to find that the core of KP is [1, 10000], so this problem is difficult to tackle by the exact algorithm in [17]. In this paper, it is easy to get an approximate solution of KP which objective value is 6395122580. The solution can be proved to be the optimal solution.

In this paper, a fast polynomial approximate solution is proposed for 0-1 knapsack problems based on the solution of its upper bound. Firstly, an upper bound is presented based on the exact *k*-item knapsack problem E-*k*KP in Section 2. Secondly, an initial solution of KP is constructed on the basis of the solution of upper bound of E-*k*KP in Section 3.1. Thirdly, the approximate solution is proposed to find the best solution in the neighborhood of the initial solution in Section 3.2. In Section 3.3, the best approximation solution is achieved by comparison with number of items changing. In Section 3.4, the calculation for the approximate solution of KP is analyzed. In Section 4, computational experiments of KP are implemented. The results show that the approximate solution proposed in this paper can achieve high accuracy in general. It implicates that the exact solution to KP is similar to the solution to the upper bound. The algorithm proposed here is a fast polynomial approximate solution to KP.

## 2. Upper Bound for KP

If there are exact *k* objects selected in the knapsack, then KP is an exact *k*-item knapsack problem E-*k*KP formulated as follows [3]:

(4) $$\begin{array}{c} \max\;f\left(x\right)=\overset{n}{\underset{i=1}{\sum }}p_{i}x_{i}\text{,}\\ s.t,\\ \overset{n}{\underset{i=1}{\sum }}w_{i}x_{i}\leq c\text{,}\\ \overset{n}{\underset{i=1}{\sum }}x_{i}=k\text{,}\\ x_{i}=0,1.i=1,2,\cdots ,n\text{.} \end{array}$$

The upper bound of E-*k*KP may be achieved by Lagrangian relaxation of capacity constraint:

(5) $$\begin{array}{c} L\left(k,\lambda \right)=\lambda c+\max\overset{n}{\underset{i=1}{\sum }}\left(p_{i}-\lambda w_{i}\right)u_{i}\text{,}\\ s.t,\\ \overset{n}{\underset{i=1}{\sum }}u_{i}=k\text{,}\\ u_{i}=0,1.i=1,2,\cdots ,n\text{.} \end{array}$$

Suppose that

(6) $$\begin{array}{c} p_{d_{k1}}-\lambda w_{d_{k1}}\geq p_{d_{k2}}-\lambda w_{d_{k2}}\geq \cdots \geq p_{d_{kn}}-\lambda w_{d_{kn}},w_{d_{kk}}\leq w_{d_{kk+1}}\text{.} \end{array}$$

Then

(7) $$\begin{array}{c} L\left(k,\lambda \right)=\lambda c+\sum_{i=1}^{k}\left(p_{d_{ki}}-\lambda w_{d_{ki}}\right). \end{array}$$

So *L*(*k*, *λ*) can be solved by sorting *p*_*i*_ − *λw*_*i*_ in descending order in *O* (*n*ln*n*) time if *λ* and *k* are fixed. The upper bound of E-*k*KP is formulated as follows:

(8) $$\begin{array}{c} B_{k}=\min_{\lambda \geq 0}L\left(k,\lambda \right)\text{.} \end{array}$$

It can be proved that *L*(*k*, *λ*) is a unimodal function of *λ* if *k* is fixed. *B*_*k*_ can be quickly solved by linear search algorithm [19]. So *B*_*k*_ can be solved in *O* (*n*ln*n*) time.

The upper bound of KP is the maximum value of *B*_*k*_:

(9) $$\begin{array}{c} B=\max_{0\leq k\leq n}B_{k}\text{.} \end{array}$$

It can be proved that *B*_*k*_ increases when *k* < *s* − 1 and decreases when *k* > *s*, where the critical item *s* satisfies.

(10) $$\begin{array}{c} 0\leq c-w_{1}-w_{2}-\cdots -w_{s-1}<w_{s}\text{.} \end{array}$$

So the upper bound of KP is

(11) $$\begin{array}{c} B=\min\;\left\{B_{s-1},B_{s}\right\}\text{.} \end{array}$$

**Example 1 .**

Consider the instance of KP listed in Table 1. The capacity is 467.8435.

Here the critical item *s* is 20. There is no feasible solution with 20 items included in the knapsack. So the upper bound *B*_20_= 0. If *k* is 19, we have the optimal ratio *λ*_19_ = 1.0003251 and the upper bound *B*_19_ = 486.9565 by (8). The solution of *L*(19, *λ*_19_) is *u*^(19)^ = (*u*_*d*_19*i*__)as follows:

(12) $$\begin{array}{c} u_{d_{19i}}=\left\{\begin{array}{cc} 1\text{,} & i\leq 19\text{,}\\ 0\text{,} & i>19\text{,} \end{array}\right.\\ \left(d_{19i}\right)=\left(2,1,4,7,5,3,10,6,9,16,8,13,18,11,26,20,21,17,14,27,28,23,12,15,19,30,25,24,22,29\right)\text{.} \end{array}$$

So the upper bound of KP is 486.9565.

We test different upper bounds of KP from Pisinger's paper [20], where the weights and profits of items are randomized. The instances listed in Table2 are tested to compare our proposed method with the existing upper bounds, such as the upper bound *U*_MT2_ proposed by Martello and Toth [21], the improved upper bound *U*_MTM_ [22] and the upper bound *U*_*k*max_ with maximum cardinality [23]. The difficult instances can be constructed as follows [20]:

- Uncorrelated instances with similar weights: Weights *w*_*i*_ are distributed in [*R*, *R* + 100] and the profits *p*_*i*_ in [1, 1000].
- Uncorrelated data instances: *p*_*i*_ and *w*_*i*_ are chosen randomly in [1, *R*].
- Weakly correlated instances: Weights *w*_*i*_ are chosen randomly in [1, *R*] and the profits *p*_*i*_ in [*w*_*i*_ − 0.1 *R*, *w*_*i*_ + 0.1 *R*] such that *p*_*i*_ ≥ 1.
- Strongly correlated instances: Weights *w*_*i*_ are distributed in [1, *R*] and *p*_*i*_ = *w*_*i*_ + 0.1*R*.
- Inverse strongly correlated instances: Profits *p*_*i*_ are distributed in [1, *R*] and *w*_*i*_ = *p*_*i*_+0.1*R*.
- Almost strongly correlated instances: Weights *w*_*i*_ are distributed in [1, *R*] and the profits *p*_*i*_ in [*w*_*i*_ + 0.098*R*, *w*_*i*_ + 0.102*R*].
- Subset sum instances: Weights *w*_*j*_ are randomly distributed in [1, *R*] and *p*_*i*_ = *w*_*i*_.
- Circle instances circle (*d*): The weights are uniformly distributed in [1, *R*] and for each weight *w* the corresponding profit is chosen as $p=d\sqrt{4wR-w^{2}}$ where *d* is 2/3.
- Profit ceiling instances pceil (*d*): The weights of the *n* items are randomly distributed in [1, *R*], and the profits are set to *p*_*i*_ = *d*⌈*w*_*i*_/*d*⌉. The parameter *d* was chosen as *d* = 3.
- Multiple strongly correlated instances mstr (*k*_1_, *k*_2_, *d*): The weights of the *n* items are randomly distributed in [1, *R*]. If the weight *w*_*i*_ is divisible by *d*, then we set the profit *p*_*i*_: = *w*_*i*_ + *k*_1_; otherwise set it to *p*_*i*_: = *w*_*i*_ + *k*_2_. We set *d*: = 6 here.

For each instance type, a series of *K* = 100 instances is performed, and the capacity is determined by (13). All the instances above are generated with data range *R* = 10^3^, 10^4^, 10^5^, 10^6^ or 10^7^.

(13) $$\begin{array}{c} c=\frac{k}{1+K}\overset{n}{\underset{i=1}{\sum }}w_{i},k=1,2,\cdots ,K\text{.} \end{array}$$

There are 100 instances for each type of KP where the capability is described as follows:

(14) $$\begin{array}{c} C=\left[\frac{k}{101}\overset{n}{\underset{i=1}{\sum }}w_{i}\right],k=1,2,\cdots ,100,n=10^{4}\text{.} \end{array}$$

The mean relative error of upper bounds to the best upper bound of KP is listed in Table 3.

From Table 3, we find that the relative error of the upper bound *B* is the minimum in general. The upper bounds of 5000 instances are carried out with different methods. More details are listed in Table 4.

From Table 4, we find that *U*_*k*max_ is the best upper bound in 2025 instances. The upper bound *B* plays an important role in obtaining the best upper bound even if *s* ≤ *k*_max_.

The upper bound can gather most items selected in the optimal solution all together. For example, KP is determined by

(15) $$\begin{array}{c} w_{i}=i,p_{i}=10i^{2}+10^{7},c=200020,i=1,2,\cdots ,10^{4}\text{.} \end{array}$$

A maximum of 631 items can be selected in the knapsack. The upper bound *B*_*s*−1_(*s*=632) and the solution *u*^(*s* − 1)^ are as follows:

(16) $$\begin{array}{c} B_{s-1}=\min_{\lambda \geq 0}\left\{\max\;\left\{\lambda c+\overset{10000}{\underset{i=1}{\sum }}\left(p_{i}-\lambda w_{i}\right)x_{i}\left|\overset{10000}{\underset{i=1}{\sum }}x_{i}=s-1,x_{i}=0,1\right.\right\}\right\}\\ =106310c+\overset{631}{\underset{i=1}{\sum }}\left(p_{i}-106310w_{i}\right)=7215794600\text{,}u_{i}=\left\{\begin{array}{cc} 1\text{,} & i=1,2,...,631\text{,}\\ 0\text{,} & 0,631<i\leq 10000\text{.} \end{array}\right. \end{array}$$

The initial solution *z*^(*s* − 1)^ is equal to *u*^(*s* − 1)^. The best solution *y*_*a*_^(*s* − 1)^ in the neighborhood ∪_*h*=1_^4^*N*(*z*^(*s* − 1)^, 2*h*) can be described as follows:

(17) $$\begin{array}{c} y_{i}=\left\{\begin{array}{cc} 1\text{,} & i=1,2,...,630,1225\text{,}\\ 0\text{,} & i\neq 1225,630<i\leq 10000\text{.} \end{array}\right. \end{array}$$

**Figure 1:**
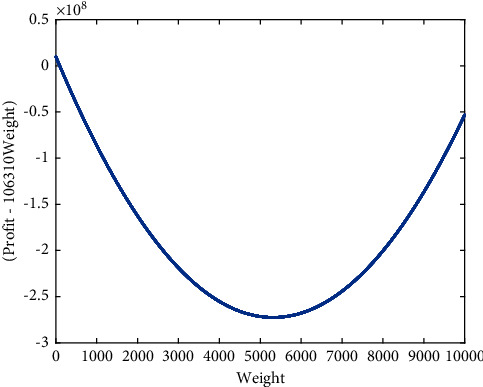
Relation between (*p*_*i*_ − 106310*w*_*i*_) and *w*_*i*_.

**Figure 2:**
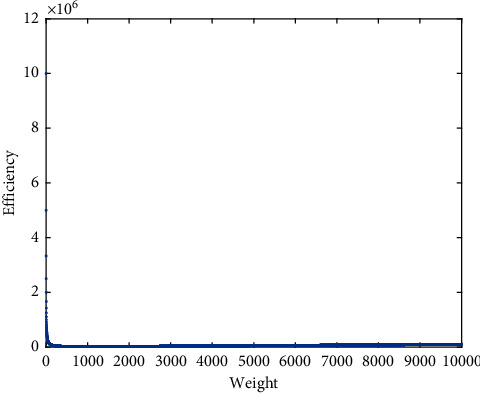
Relation between (*p*_*i*_/*w*_*i*_) and *w*_*i*_.

It is obviously that there is little difference between *z*^(*s* − 1)^ and *y*_*a*_^(*s* − 1)^. Relation between (*p*_*i*_ − 106310*w*_*i*_) and *w*_*i*_ is displayed in Figure 1. Relation between *p*_*i*_/*w*_*i*_ and *w*_*i*_ is displayed in Figure 2. It is found that (*p*_*i*_ − 106310*w*_*i*_) changes more dramatically than *p*_*i*_/*w*_*i*_. It makes solution to KP easy.

From Figure 1, we can see that (*p*_*i*_ − 106310*w*_*i*_) of all items selected are larger, while the others are smaller. It is easy to get a better solution on the basis of (*p*_*i*_ − 106310*w*_*i*_) than that on the basis of efficiency *p*_*i*_/*w*_*i*_.

## 3. Approximate Solutions

The approximate solution to E-*k*KP is a solution to KP. We obtain the best approximate solution to KP by comparison to approximate solutions for E-*k*KP where *k* is near to the critical item *s*. In order to achieve a better solution to E-*k*KP, we firstly obtain an initial solution on the basis of the solution to the upper bound of E-*k*KP. Then the initial solution is developed by local search in the neighborhood of the initial solution. At last, the approximate solution to KP is the best approximate solution to E-*k*KP with various *k*. The upper bound of E-*k*KP is used to decrease calculation. The solution to the upper bound of E-*k*KP and the upper bound make key contribution in FPTAS to KP.

### 3.1. Initial Solution to E-*k*KP

Let *u*^(*k*)^ = (*u*_*d*_*ki*__) be an optimal solution to the upper bound *B*_*k*_. We may obtain an initial solution to E-*k*KP on the basis of *u*^(*k*)^. The initial solution *z*^(*k*)^ to E-*k*KP is determined by

(18) $$\begin{array}{c} u_{d_{ki}}=\left\{\begin{array}{cc} 1\text{,} & i\leq k\text{,}\\ 0\text{,} & k<i\leq n\text{,} \end{array}\right.\\ z_{d_{ki}}=\left\{\begin{array}{c} u_{d_{ki}},i\neq k,k+1\text{,}\\ u_{d_{ki}},w_{d_{kk+1}}>c-\overset{k-1}{\underset{j=1}{\sum }}w_{d_{kj}},i=k,k+1\text{,}\\ 1-u_{d_{ki}},w_{d_{kk}}>c-\overset{k-1}{\underset{j=1}{\sum }}w_{d_{kj}},i=k,k+1\text{,}\\ u_{d_{ki}},c-\overset{k-1}{\underset{j=1}{\sum }}w_{d_{kj}}\geq \max\;\left\{w_{d_{kk}},w_{d_{kk+1}}\right\},p_{d_{kk}}\geq p_{d_{kk+1}},i=k,k+1\text{,}\\ 1-u_{d_{ki}},c-\overset{k-1}{\underset{j=1}{\sum }}w_{d_{kj}}\geq \max\;\left\{w_{d_{kk}},w_{d_{kk+1}}\right\},p_{d_{kk}}<p_{d_{kk+1}},i=k,k+1\text{.} \end{array}\right. \end{array}$$

In Example 1, *z*^(19)^ is an initial solution and its objective value is

(19) $$\begin{array}{c} f\left(z^{\left(19\right)}\right)=473.1749\\ Zd19i=\left\{\begin{array}{c} 1,i\leq 19\\ 0,i>19 \end{array}\right.\\ \left(d_{19i}\right)=\left(2,1,4,7,5,3,10,6,9,16,8,13,18,11,26,20,21,17,14,27,28,23,12,15,19,30,25,24,22,29\right)\text{.} \end{array}$$

### 3.2. Approximate Solution to E-*k*KP

Approximate solution to E-*k*KP is the best solution in the neighborhood of *z*^(*k*)^. Let *N*(*z*^(*k*)^, 2*h*) be a neighborhood of *z*^(*k*)^ that is defined by

(20) $$\begin{array}{c} N\left(z^{\left(k\right)},2h\right)=\left\{y^{\left(k\right)}=\left(y_{d_{ki}}\right)\left|\overset{n}{\underset{i=1}{\sum }}\left|y_{d_{ki}}-z_{d_{ki}}\right|\right.=2h,\overset{n}{\underset{i=1}{\sum }}y_{d_{ki}}=k,y_{d_{ki}}=0,1\right\}\text{.} \end{array}$$

It is obvious that the size of *N*(*z*^(*k*)^, 2*h*) increases with *h*. But it is unnecessary to take into account all elements of *N*(*z*^(*k*)^, 2*h*).  Algorithm 1 is a fast algorithm for searching the best solution in *N*(*z*^(*k*)^, 2*h*).

In order to decrease the calculation for the approximate solution to KP, *N*(*z*^(*k*)^, 2*h*) is redefined by (21) and Step 1 in Algorithm 1 is modified correspondingly.

(21) $$\begin{array}{c} N\left(z^{\left(k\right)},2h\right)=\left\{y^{\left(k\right)}=\left(y_{d_{ki}}\right)\left|\sum_{i=L_{kh}}^{U_{kh}}\left|y_{d_{ki}}-z_{d_{ki}}\right|\right.=2h,\sum_{i=1}^{n}y_{d_{ki}}=k,y_{d_{ki}}=0,1\right\}\text{,} \end{array}$$

where

(22) $$\begin{array}{c} L_{kh}=\left\{\begin{array}{c} 1,h=1\\ \max\;\left\{k-100,1\right\},h=2\\ \max\;\left\{1,k-50\right\},h=3\\ \max\;\left\{k-30,1\right\},h=4 \end{array}\right.\\ U_{kh}=\left\{\begin{array}{c} n,h=1\\ \min\;\left\{99+k,n\right\},h=2\\ \min\;\left\{49+k,n\right\},h=3\\ \min\;\left\{29+k,n\right\},h=4 \end{array}\right.\\ s-5\leq k\leq s+4\text{.} \end{array}$$

From (21), we know that the size of *N*(*z*^(*k*)^, 2*h*)(*h* = 1,2,3,4) is limited. In order to search for the best solution, it is unnecessary to seek all solutions in *N*(*z*^(*k*)^, 2*h*). Let

(23) $$\begin{array}{c} r_{w}=c-w_{d_{k1}}-w_{d_{k2}}-\cdots -w_{d_{kk}}\\ \left\{\left(p_{s1},w_{s1}\right)|p_{s1}=p_{d_{ki_{1}}}+\cdots +p_{ki_{h}},w_{s1}=w_{d_{ki_{1}}}+\cdots +w_{d_{ki_{h}}}+r_{w},\max\left\{1,L_{kh}\right\}\leq i_{1}<\cdots <i_{h}\leq k\right\}\\ \left\{\left(p_{s2},w_{s2}\right)|p_{s2}=p_{d_{kj_{1}}}+\cdots +p_{d_{kj_{h}}},w_{s2}=w_{d_{kj_{1}}}+\cdots +w_{d_{kj_{h}}},k<j_{1}<\cdots <j_{h}\leq \min\left\{n,U_{kh}\right\}\right\}. \end{array}$$

The best approximate solution corresponds to max {*p*_*s*2_ − *p*_*s*1_|*w*_*s*2_ ≤ *w*_*s*1_}, so the calculation is *O* (*n*ln*n*) time when *h* equals to 1. A better solution is generated if the profit sum of *h* items selected in the knapsack is less than that of *h* items not selected in the knapsack, and the capacity constraint still holds at the same time. On the other hand, the calculation decreases via variable reduction in practice.

In Example 1, let the approximate solution *x*_*a*_ equal to *z*^(19)^ and objective value *f*_*a*_ equal to *f*(*z*^(19)^) firstly, and then the approximate solution *x*_*a*_ is updated by the best solution *y*_*a*_^(19)^ in *N*(*z*^(19)^, 2) if possible.

(24) $$\begin{array}{c} f\left(y_{a}^{\left(19\right)}\right)=486.1884>f_{a}=473.1749\\ yd_{19i}=\left\{\begin{array}{c} z_{d_{19i}},i\leq 30,i\neq 12,22\\ 1-z_{d_{19i}},i=12,22 \end{array}\right.\\ f_{a}\leftarrow f\left(y_{a}^{\left(19\right)}\right),x_{a}\leftarrow y_{a}^{\left(19\right)}\text{.} \end{array}$$

Similarly, *x*_*a*_ is replaced by the best solution *y*_*a*_^(19)^ in *N*(*z*^(19)^, 4) if possible.

(25) $$\begin{array}{c} f\left(y_{a}^{\left(19\right)}\right)=486.9426>f_{a}=486.1884\\ yd_{19i}=\left\{\begin{array}{c} 1,i=1,2,...,21,i\neq 13,17\\ 0,i=13,17,22,...,30 \end{array}\right.\\ f_{a}\leftarrow f\left(y_{a}^{\left(19\right)}\right),x_{a}\leftarrow y_{a}^{\left(19\right)}\text{.} \end{array}$$

There is no better solution of KP in *N*(*z*^(19)^, 6) and *N*(*z*^(19)^, 8). We get an approximate solution *y*_*a*_^(19)^ with 19 items selected and the approximate objective value is 486.9426.

### 3.3. Approximation Algorithm of KP

Let *x*_*a*_ describe the best approximate solution to KP in (26).

(26) $$\begin{array}{c} x_{a}=\underset{k}{argmax}f\left(y_{a}^{\left(k\right)}\right)=\underset{k}{argmax}\max_{1\leq h\leq 4}\left\{f\left(y^{\left(k\right)}\right)\left|y^{\left(k\right)}\in N\left(z^{\left(k\right)},2h\right)\right.\right\}\text{.} \end{array}$$

We can obtain the approximate solution *x*_*a*_ of KP by Algorithm 2.

In Example 1, we obtain *f*_*a*_=486.9426 when *k* = 19.

(27) $$\begin{array}{c} B_{20}=0,B_{18}=485.9582<f_{a}=486.9426\text{.} \end{array}$$

The best approximate solution *x*_*a*_ = (*x*_*d*_19*i*__) for KP and its objective value are as follows:

(28) $$\begin{array}{c} f_{a}=486.9426\text{.} \end{array}$$

It may be proved that *x*_*a*_ is the optimal solution to KP in Example 1.

### 3.4. Calculation Analysis

Approximation algorithms for KP based on upper bound of E-*k*KP runs in polynomial time. Calculation for the upper bound of E-*k*KP is less than *O* (*n*ln*n*) for a given E-*k*KP. We obtain the approximate solution to KP on the basis of the upper bound of E-*k*KP where *k* is an integer close to *s*. For a given *k*, the initial solution is determined by (13). It takes *O* (*n*ln*n*) time to develop the initial solution in the neighborhood *N*(*z*^(*k*)^, *h*) (*h* = 1, 2,…, 4) defined by (21). Hence, it takes at most *O* (*n*ln*n*) time to achieve the approximate solution to E-*k*KP. Generally, we obtain the best approximate solution for KP among the approximate solutions for E-*k*KP where *k* is near the critical item *s*. Hence, the calculation for approximate solution to KP is less than *O* (*n*ln*n*).

In Algorithm 1, we search for the best solution in *N*(*z*^(*k*)^, *h*) defined by (21). In order to develop the approximate solution quickly, the weight sum of *h* items is sorted in ascending order firstly, and then the profit sum of *h* items selected in the knapsack is compared with the profit sum of *h* items not selected. So the storage needed in Algorithm 1 is *O*(*n*) when *h* is fixed at 1, 2, 3, or 4. In Algorithm 2, we have the best solution to KP by comparison to the best approximate solution to E-*k*KP where *k* is an integer close to *s*. So the storage of the approximate algorithm for KP is *O*(*n*).

### 3.5. Accuracy Analysis

All feasible solutions to KP are in the search scope of approximation algorithm with changing *k* and *h*. So approximation algorithm for KP is an *ε*-approximation algorithm. From one aspect, weights, profits, capacity and size of KP have influence on the accuracy of an approximation algorithm. From another aspect, the search scope of the approximation algorithm has influence on its accuracy as well. Better solution is usually with more calculation. The exact algorithm for KP may be explored on the basis of the branch and bound algorithm here. Intensive research will be carried out in the future.

## 4. Computational Experiments

By Algorithm 2, we get the approximate solutions to KP listed in Table 5. The results are listed in Table 6. The optimal value listed in Table 6 is carried out by combo [24].

From Table 6, we find that the approximate solutions are almost the optimal solutions in 18 instances. It implicates that the approximate algorithm proposed here achieves high precision in solving KP.

In Table 7 lists the experimental results of the solutions for KP listed in Table 2 by Algorithm 2. The upper bound listed in Table 4 is carried out by equation (11) in Section 2.

From Table 7, we see that the relative average error of 100 instances is almost less than 0.0001. The experiment result shows that the approximate algorithm proposed here can achieve high accuracy.

From Table 8, we find that the upper bound of E-*k*KP makes key contribution in FPTAS. Firstly, the initial solution constructed on the basis of the solution to the upper bound of E-*k*KP is similar with the optimal solution. For example, there are only 4 elements different between the initial solution and the optimal solution to E-*k*KP where *k* equals to 19, while there are 8 elements different between the initial solution constructed by efficiency and the optimal solution to KP in Table 8. Secondly, the differences between the initial solution and the solution to the upper bound of E-*k*KP are near to the item *d*_*kk*_. It is to say, we search the optimal solution to KP in a core with small size on the basis of the solution to the upper bound of E-*k*KP. While the optimal solution to KP in a core with large size on the basis of the solution to the upper bound of Dantzig. So, algorithms proposed here is easy to get approximate solution to KP.

Furthermore, no better solution of E-*k*KP exists when the objective value *f*_*a*_ of the approximate solution achieved before is larger than the upper bound *B*_*k*_. So we only search solution of E-*k*KP with upper bound larger than *f*_*a*_ which is in the neighborhood of the optimal solution to the upper bound of E-*k*KP. The candidate strategy makes the search in polynomial time and the solution with high accuracy in Algorithm 1. The upper bound of E-*k*KP plays important role in decreasing calculation of the approximate solution of E-kKP in Algorithm 2 as well.

## 5. Conclusion

It is still difficult to obtain the exact solution for large scale 0-1 knapsack problem directly. Here a fast polynomial approximate solution is proposed on the basis of the upper bound for KP. The exact solution to KP is in the neighborhood of the solution to the upper bound for E-*k*KP. Therefore, it is possible to find an approximation with high accuracy in the neighborhood of the solution to the upper bound for E-*k*KP where *k* is near to the critical item *s*. All in all, as the basis of fast exact algorithm for KP, it is important to obtain an approximate solution and the upper bound for KP. In order to obtain a fast exact solution to KP, more intensive research on variables reduction need be conducted in the future.

## Figures and Tables

**Algorithm 1:**
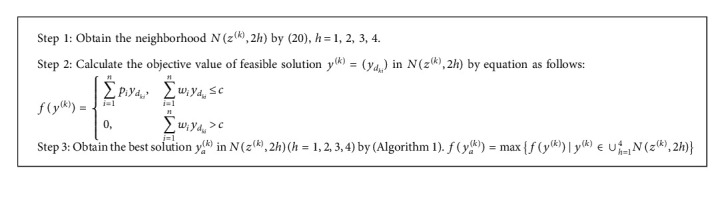
Approximate solution to (E-*k*KP).

**Algorithm 2:**
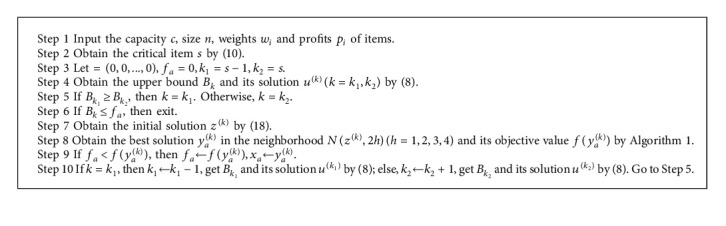
Approximate Algorithm for (KP).

**Table 1:** Weight and profit of items.

*i*	*w*_*i*_	*p*_*i*_
1	1.7856	2.7924
2	4.877	5.8853
3	6.3493	7.3521
4	7.0943	8.0992
5	7.8807	8.885
6	8.5593	9.5616
7	13.9249	14.9319
8	19.6114	20.6148
9	21.0881	22.0925
10	24.2688	25.2767
11	27.3441	28.3472
12	31.618	32.6189
13	32.7739	33.7797
14	32.787	33.7898
15	33.9368	34.9379
16	37.1566	38.1662
17	37.887	38.892
18	39.6104	40.6175
19	40.014	41.0159
20	40.7362	41.7432
21	42.4565	43.463
22	45.2896	46.2899
23	45.6688	46.6693
24	45.7868	46.7932
25	46.6997	47.7013
26	47.8583	48.866
27	47.8753	48.8848
28	47.9746	48.9822
29	48.2444	49.2448
30	48.5296	49.5335

**Table 2:** Weights and profits of knapsack problems from Pisinger's paper [20].

No.	(*w*_*i*_, *p*_*i*_) (*i* = 1, 2,…, 10^4^)	No.	(*w*_*i*_, *p*_*i*_) (i = 1, 2,…, 10^4^)
1	([*R*+100*u*_*i*_], ⌈1000*v*_*i*_⌉)	2	(⌈*Ru*_*i*_⌉, ⌈*Rv*_*i*_⌉)
3	([*Ru*_*i*_], [*R*(*u*_*i*_+0.2*v*_*i*_ − 0.1)])	4	([*Ru*_*i*_], [*Ru*_*i*_+0.1*R*])
5	([*Rv*_*i*_+0.1*R*], [*Rv*_*i*_])	6	([*Ru*_*i*_+0.098*R*], [*R*(*u*_*i*_+0.102*R*)])
7	(⌈*Ru*_*i*_⌉, ⌈*Ru*_*i*_⌉)	8	$\left(\left[Ru_{i}\right],\left[2R/3\sqrt{u_{i}\left(4-u_{i}\right)}\right]\right)$
9	([*Ru*_*i*_], ⌈*Ru*_*i*_/3⌉)	10	$\begin{array}{c} \left(\left[Ru_{i}\right],\left[Ru_{i}+0.3R\right]\right),\left[Ru_{i}\right]\%6=0\text{;}\\ \left(\left[Ru_{i}\right],\left[Ru_{i}+0.2R\right]\right),\left[Ru_{i}\right]\%6\neq 0 \end{array}$

^*∗*^ *Note*. *u*_*i*_ and *v*_*i*_ are uniformly distributed in [0,1]. *R* = 10^3^, 10^4^, 10^5^, 10^6^ or 10^7^.

**Table 3:** Mean relative error of upper bounds to the best upper bound of (KP) (ppm).

(No, *R*)	*U*_MT2_	*U*_MTM_	*U*_*k*max_	*B*	(No, *R*)	*U*_MT2_	*U*_MTM_	*U*_*k*max_	*B*
(1, 10^3^)	4.6696	4.6696	4.6869	0	(2, 10^3^)	0.0138	0.0031	0.0421	0.0174
(1, 10^4^)	91.413	91.3869	81.4422	0	(2, 10^4^)	0.0345	0.0075	0.0465	0.0189
(1, 10^5^)	60.2607	60.2542	43.2765	0	(2, 10^5^)	0.0194	0.005	0.0351	0.0164
(1, 10^6^)	61.0366	61.0366	0	0	(2, 10^6^)	0.0258	0.0057	0.0391	0.0152
(1, 10^7^)	61.3372	61.3372	0	0	(2, 10^7^)	0.0274	0.0069	0.0466	0.018
(3, 10^3^)	0.0116	0.0049	0.0179	0	(4, 10^3^)	28.7876	28.7876	0	0
(3, 10^4^)	0.0031	0	0.0196	0.0044	(4, 10^4^)	34.656	34.6518	0	0
(3, 10^5^)	0.0148	0.0018	0.027	0.016	(4, 10^5^)	31.5535	31.5456	0	0
(3, 10^6^)	0.0114	0.0016	0.0166	0.0071	(4, 10^6^)	27.5082	27.5001	0	0
(3, 10^7^)	0.0066	0.0022	0.0105	0.0031	(4, 10^7^)	25.7493	25.7467	0	0
(5, 10^3^)	38.9054	38.9054	38.9054	0	(6, 10^3^)	0	0	0	0
(5, 10^4^)	0	0	0	0	(6, 10^4^)	0	0	0	0
(5, 10^5^)	0	0	0	0	(6, 10^5^)	0	0	0	0
(5, 10^6^)	0	0	0	0	(6, 10^6^)	0	0	0	0
(5, 10^7^)	0	0	0	0	(6, 10^7^)	0	0	0	0
(7, 10^3^)	0	0	0	0	(8, 10^3^)	0	0	0	0
(7, 10^4^)	0	0	0	0	(8, 10^4^)	0	0	0	0
(7, 10^5^)	0	0	0	0	(8, 10^5^)	22.8602	22.8602	0	0
(7, 10^6^)	0	0	0	0	(8, 10^6^)	0	0	0	0
(7, 10^7^)	0	0	0	0	(8, 10^7^)	0	0	0	0
(9, 10^3^)	0	0	0	0	(10, 10^3^)	0	0	0	0
(9, 10^4^)	0	0	0	0	(10, 10^4^)	0	0	0	0
(9, 10^5^)	16.1344	16.1344	0	0	(10, 10^5^)	0	0	0	0
(9, 10^6^)	30.1447	30.1447	0	0	(10, 10^6^)	0	0	0	0
(9, 10^7^)	8.9156	8.9156	8.9156	0	(10, 10^7^)	0	0	0	0

^*∗*^The best upper bound is min{*U*_MT2_, *U*_MTM_, *U*_kmax_, *B*} in this paper.

**Table 4:** Sum of the best upper bounds in 5000 instances.

Upper bound	Sum of the best upper bound	Percentage of the best upper bound (%)	Sum of the best upper bounds equal to *B*
*U*_MT2_	1520	30.40	1495
*U*_MTM_	1925	38.50	1548
*U*_*k*max_	2025	40.50	2025
*B*	4623	92.46	4623

**Table 5:** 0/1 Knapsack problems.

No.	(*w*_*i*_*, p*_i_) (*i* = 1, 2,…, 10000)	*C*
1	$\left(i,\sqrt{i}\right)$	2.6*e* + 07
2	(*i*, ln *i*+1)	2.7*e* + 07
3	(*i*, tan (*πi*/30000))	2.8*e* + 07
4	(*i*, 100+10^−4^*i*^2^)	2.9*e* + 07
5	(*i*, 0.01*i* − 5600)^2^+1)	3.0*e* + 07
6	(*i*, *i*+tan (*πi*/30000))	3.1*e* + 07
7	(*i*, tan (*πi*/30000)+0.001*i*^2^)	3.2*e* + 07
8	(*i*, 5000+10^−6^*i*^3^)	3.3*e* + 07
9	(*i*, tan (*πi*/30000)+10^−6^*i*^3^)	3.4*e* + 07
10	(*i*, arctan (0.01*i*)+1)	3.5*e* + 07
11	(*i*, 3 − lncos(10^−4^*i*))	3.6*e* + 07
12	(*i*, arctan (10^−4^*i*^2^)+1)	3.7*e* + 07
13	(*i*, sin *e*^10^−4^*i*^+10^−4^*i*^2^)	3.8*e* + 07
14	(*i*, sin *e*^10^−4^*i*^+10^−2^*i*)	3.9*e* + 07
15	(*i*, (1/*i*))	4.0*e* + 07
16	(*i*, (*e*^10^−4^*i*^+*e*^−10^−4^*i*^/sin (10^−4^*i*)))	4.1*e* + 07
17	(*i*, *i*^2^)	4.2*e* + 07
18	(*i*, *i*+2+*ε*_*i*_), *ε*_*i*_ ~ [0,0.003]	4.3*e* + 07

**Table 6:** Approximate value and optimization value of 0/1 Knapsack problems.

No.	Approximate value	Optimal value
1	4.082159596963170*e* + 05	4.082159596963174*e* + 05
2	6.541219347026785*e* + 04	6.541219347026781*e* + 04
3	4.102160742890480*e* + 03	4.102160742890481*e* + 03
4	2.462154058619999*e* + 07	2.462154058620000*e* + 07
5	8.338180285999998*e* + 08	8.338180286000001*e* + 08
6	3.100447382723671*e* + 07	3.100447382723672*e* + 07
7	2.613912845586523*e* + 07	2.613912845586526*e* + 07
8	2.232528710024500*e* + 09	2.232528710024500*e* + 09
9	2.244286940564110*e* + 09	2.244286940564110*e* + 09
10	2.096538351264514*e* + 04	2.096538351264514*e* + 04
11	2.655627458756987*e* + 04	2.655627458756988*e* + 04
12	2.189122733748200*e* + 04	2.189122733748204*e* + 04
13	2.942021969836180*e* + 07	2.942021969836181*e* + 07
14	3.981146835799920*e* + 05	3.981146835799920*e* + 05
15	9.675897955855723	9.675897955855723
16	1.997554888239176*e* + 05	1.997554888239177*e* + 05
17	3.120269950000000*e* + 11	3.120269950000000*e* + 11
18	4.301855990805434*e* + 07	4.301855990837396*e* + 07

**Table 7:** Relative average error to the upper bound (ppm).

*R*	No. 1	No. 2	No. 3	No. 4	No. 5	No. 6	No. 7	No. 8	No. 9	No. 10
10^3^	5.6579	2.7082	1.6469	0.4003	0	1.2883	0.0573	100.5351	41.0547	24.8855
10^4^	0.3227	1.5208	0.7258	0.0157	0.0132	0.4878	0	46.7007	0.131	21.4483
10^5^	0.0591	1.1447	0.4877	0.0141	0.0444	0.2357	0.0044	43.6594	0.0148	19.1698
10^6^	0.0577	1.1524	0.6731	0.0185	0.0328	0.246	0.0047	50.0126	0.0014	19.21
10^7^	0.0510	1.205	0.5800	0.0229	0.0192	0.1975	0.0034	49.9764	0.0001	14.8858

**Table 8:** Relation between (*p*_*i*_-1.0003121*w*_*i*_) and the optimal solution (*x*_*i*_^opt^) in Example 1.

*i*	*w*_*i*_	*p*_*i*_	(*p*_*i*_/*w*_*i*_)	*e*_*j*_	*p*_*i*_ − 1.0003121*w*_*i*_	*d*_19l_	*x*_*i*_^opt^	*x*_*i*_^*ei*^	*z*_*i*_^(19)^
2	4.877	5.8853	1.20674595	2	1.006714385	1	1	1	1
1	1.7856	2.7924	1.563844086	1	1.006219464	2	1	1	1
4	7.0943	8.0992	1.141648929	4	1.002593494	3	1	1	1
7	13.9249	14.9319	1.072316498	7	1.002472723	4	1	1	1
5	7.8807	8.885	1.127437918	5	1.001737819	5	1	1	1
3	6.3493	7.3521	1.15793867	3	1.000735709	6	1	1	1
10	24.2688	25.2767	1.04153069	10	1.000009703	7	1	1	1
6	8.5593	9.5616	1.117100697	6	0.999517192	8	1	1	1
9	21.0881	22.0925	1.047628757	9	0.997543816	9	1	1	1
16	37.1566	38.1662	1.027171485	16	0.997519609	10	1	1	1
8	19.6114	20.6148	1.051164119	8	0.997023922	11	1	1	1
13	32.7739	33.7797	1.030689054	13	0.995144517	12	1	1	1
18	39.6104	40.6175	1.025425141	18	0.994221827	13	0	1	1
11	27.3441	28.3472	1.03668433	11	0.994209859	14	1	1	1
27	47.8753	48.8848	1.02108603	26	0.993934735	15	1	0	1
20	40.7362	41.7432	1.024720028	20	0.993755806	16	1	0	1
21	42.4565	43.463	1.023706617	21	0.9926965	17	0	0	1
17	37.887	38.892	1.026526249	17	0.992682141	18	1	1	1
26	47.8583	48.866	1.021055909	27	0.992140262	19	1	0	0
14	32.787	33.7898	1.030585293	14	0.992140258	20	1	1	1
28	47.9746	48.9822	1.021002781	28	0.99200245	21	1	0	0
24	45.7868	46.7932	1.021980134	23	0.99151375	22	0	0	0
12	31.618	32.6189	1.031656019	12	0.990620324	23	0	1	0
15	33.9368	34.9379	1.029498951	15	0.990066434	24	0	1	0
19	40.014	41.0159	1.025038736	19	0.988890608	25	0	1	0
30	48.5296	49.5335	1.020686344	30	0.988122008	26	0	0	0
25	46.6997	47.7013	1.021447675	25	0.986416947	27	0	0	0
23	45.6688	46.6693	1.021907736	24	0.985652114	28	0	0	0
22	45.2896	46.2899	1.022086748	22	0.9855754	29	0	0	0
29	48.2444	49.2448	1.020736085	29	0.984714732	30	0	0	0

^*∗*^ *p*_*d*_19*l*__ − 1.0003121*w*_*d*_19*l*__ ≥ *p*_*d*_19*l*+1__ − 1.0003121*w*_*d*_19*l*+1__, (*p*_*e*_*j*__/*w*_*e*_*j*__) ≥ (*p*_*e*_*j*+1__/*w*_*e*_*j*+1__), *j*, *l*=1,2, ⋯, 29. (*x*_*i*_^*ei*^) is an initial solution constructed in descending order of efficiency.

## Data Availability

All data inside the manuscript have been specified clearly in the manuscript.
